# Supplementary material for: Targeted Modulation of Chicken Genes In Vitro Using CRISPRa and CRISPRi Toolkit
Source: Genes (Basel). 2023 Apr 13;14(4):906. doi: 10.3390/genes14040906 (PMC10137795; doi:10.3390/genes14040906)
Supplement: Supplementary file 1 [file genes-14-00906-s001.zip › genes-2293299-supplementary.pdf]

Supplementary Materials

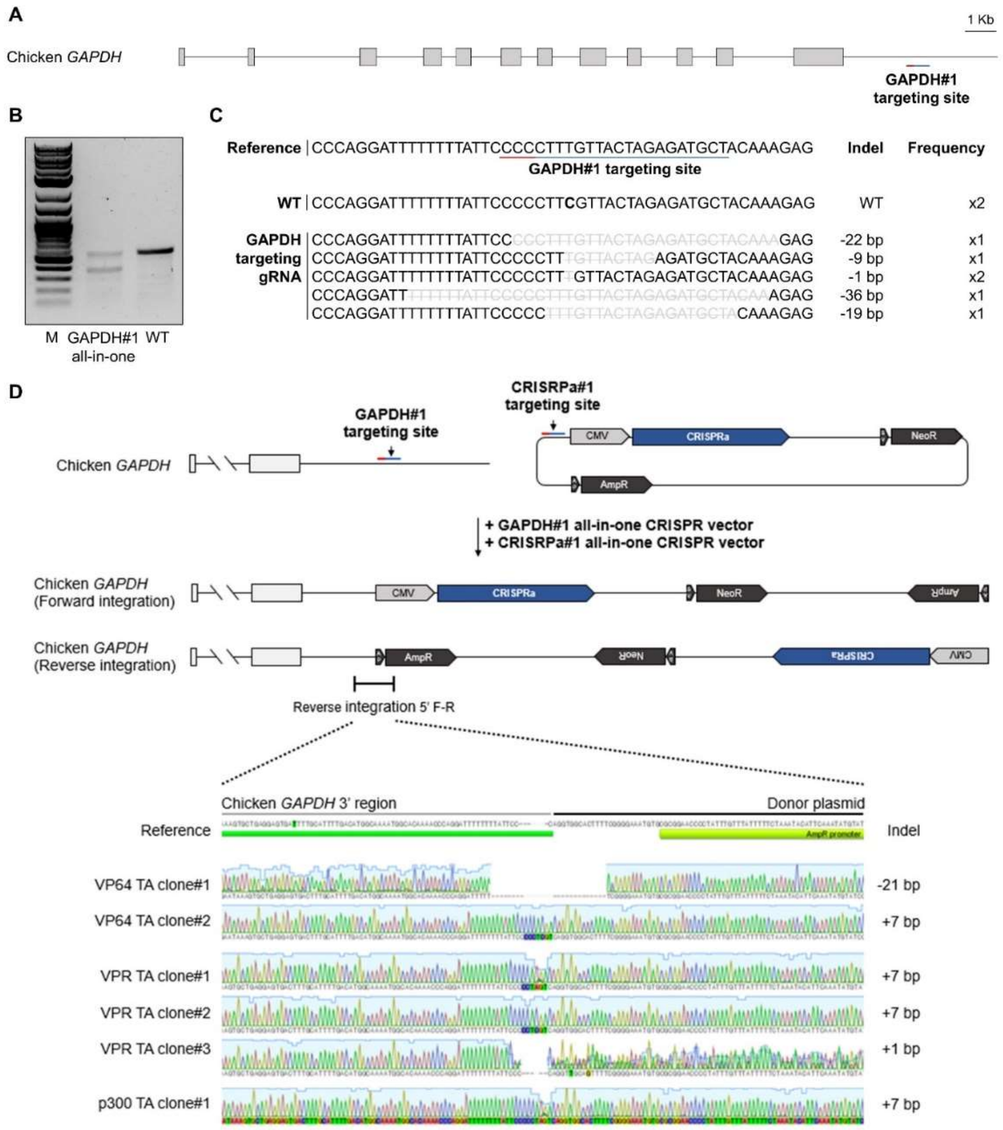

Figure S1. Targeted gene insertion of CRISPRa vectors into the 3' region of chicken *GAPDH* gene. (**A**) Gene structure of chicken GAPDH gene. Scale bar, 1 kb. (**B**,**C**) T7E1 assay and sequencing analysis of DF-1 cells transfected with the all-in-one CRISPR vector targeting the 3' region of chicken *GAPDH* gene (GAPDH#1). (**D**) Schematic representation of CRISPR/Cas9-NHEJ–mediated CRISPRa vector integration and genomic DNA analysis of targeted gene insertion in chicken DF-1 cells by knock-in–specific PCR and sanger sequencing analysis. Introduction of the donor plasmids containing CRISPRa components and two all-in-one CRISPR vectors targeting CRISPRa vectors (CRISPRa#1) and GAPDH#1 for targeted gene insertion. Blue bars indicate gRNA recognition sequences and red bars indicate protospacer adjacent motif (PAM) sequences. Nucleotide sequences of chicken genomic DNA and the donor plasmid are shown.

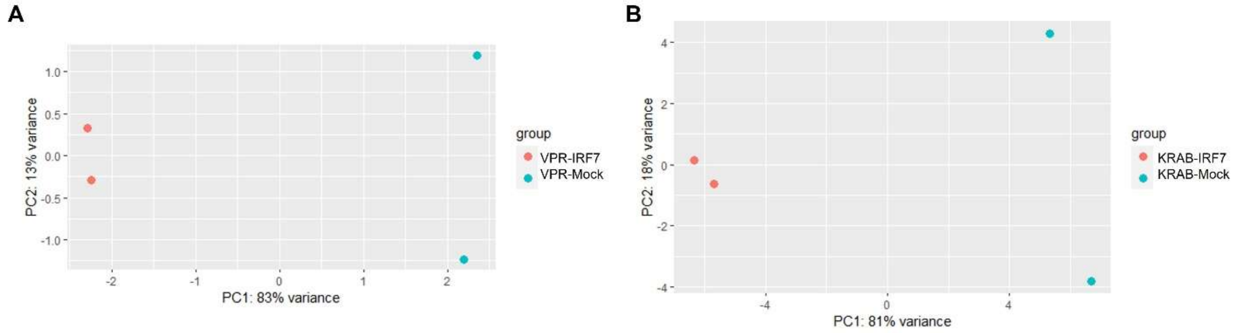

Figure S2. Principal component analysis (PCA) of bulk RNAseq libraries. (**A**) PCA plot of VPR IRF7 activation and VPR mock cells. (**B**) PCA plot of KRAB IRF7 repression and KRAB mock cells.

Supplementary Table

Table S1. Oligos used in this study.

Uses	Name	Sequence (5'–3')
GAPDH targeting gRNA construction	GAPDH gRNA#1 F	CACCGAGCATCTCTAGTAACAAAGG
	GAPDH gRNA#1 R	AAACCCTTTGTTACTAGAGATGCTC
CRISPRa vector target ing gRNA construction	CRISPRa gRNA#1 F	CACCGTCTCCCGATCCGTCGACGTC
	CRISPRa gRNA#1 F	AAACGACGTCGACGGATCGGGAGAC
CRISPRa components confirm	VP64 qRT F	GACGCATTGGACGATTTTGATC
	VP64 qRT R	CAGCATGTCCAGGTCGAAATC
	VPR qRT F	GGCACACTGTCTGAAGCTCT
	VPR qRT R	CCTCGGGGTATTCCATCAGC
	P300 qRT F	GTCAACCTGTGGACCCTCAG
	P300 qRT R	GACCTCAGAGAGCTTGGAGC
CRISPRi components confirm	dCas9 qRT F	CATCAGGGAGCAGGCAGAAA
	dCas9 qRT R	GATGAATCAGTGTGGCGTCC
	KRAB qRT F	CACGTGAGGAGTGGAAATTGC
	KRAB qRT R	GTTCTTCCCCCTTTTCGAGC
	MeCP2 F	GGTCATCAAGCGACCTGGAA
	MeCP2 R	TTTGACCTCGATGGACACGG
Knock-in confirm	GAPDH knock-in F	GTGTTGGAGGGCTGTGACTG
	GAPDH knock-in R	ATAATACCGCGCCACATAGC
gRNA expressing vector construction	IRF7 gRNA1 F	CACCGGGGATATCCGCACTACGCGG
	IRF7 gRNA1 R	AAACCCGCGTAGTGCGGATATCCCC
	IRF7 gRNA2 F	CACCGGAAACTGAAACCGCTCCGGT
	IRF7 gRNA2 R	AAACACCGGAGCGGTTTCAGTTTCC
	IRF7 gRNA3 F	CACCGGTGCGGAAGCCTCGGAGCCA
	IRF7 gRNA3 R	AAACTGGCTCCGAGGCTTCCGCACC

	IRF7 gRNA4 F	CACCGGCTGACCGTGCCGCCCCGCA
	IRF7 gRNA4 R	AAACTGCGGGGCGGCACGGTCAGCC
	IRF7 gRNA5 F	CACCGGGTCCGGGTCGATCCAGCAG
	IRF7 gRNA5 R	AAACCTGCTGGATCGACCCGGACCC
	PPARG gRNA1 F	CACCGCGTTCGCTCTTCGAACGCCC
	PPARG gRNA1 R	AAACGGGCGTTCGAAGAGCGAACGC
	PPARG gRNA2 F	CACCGCCGAGGGGCGAGCTCGCGCC
	PPARG gRNA2 R	AAAC GGCGCGAGCTCGCCCCTCGGC
	PPARG gRNA3 F	CACCGCGGTGCCTGGCCGGTAGGAT
	PPARG gRNA3 R	AAACATCCTACCGGCCAGGCACCGC
	HMGA1 gRNA1 F	CACCGTCTGAGAGCGTGAAGAAGGG
	HMGA1 gRNA1 R	AAACCCCTTCTTCACGCTCTCAGAC
	HMGA1 gRNA2 F	CACCGGCACCTCCAACCGACCCTAC
	HMGA1 gRNA2 R	AAACGTAGGGTCGGTTGGAGGTGCC
	HMGA1 gRNA3 F	CACCGACGCGCTGGAAATTAAAGTA
	HMGA1 gRNA3 R	AAACTACTTTAATTTCCAGCGCGTC
	SMARCB1 gRNA1 F	CACCGAGGGCGTCGGGTCGGTGTTG
	SMARCB1 gRNA1 R	AAACCAACACCGACCCGACGCCCTC
	SMARCB1 gRNA2 F	CACCGCGTACAAACGTTGACTCCCG
	SMARCB1 gRNA2 R	AAACCGGGAGTCAACGTTTGTACGC
	SMARCB1 gRNA3 F	CACCGGCGGGCGGCAGGTAGAAAAG
	SMARCB1 gRNA3 R	AAACCTTTTCTACCTGCCGCCCGCC
	MOCK gRNA1 F	CACCGGTGCGTTTCGTCTATACGCC
	MOCK gRNA1 R	AAACGGCGTATAGACGAAACGCAC C
	MOCK gRNA2 F	CACCGCGGTAACACGTCTATACCGG
	MOCK gRNA2 R	AAACCCGGTATAGACGTGTTACCGC
	MOCK gRNA3 F	CACCGGATCAGCTACGATCCGCCGG
	MOCK gRNA3 R	AAACCCGGCGGATCGTAGCTGATCC
qRT-PCR	GAPDH cDNA F	GGTGGTGCTAAGCGTGTTAT
	GAPDH cDNA R	ACCTCTGCCATCTCTCCACA
	IRF7 cDNA F	GAAAGCCACCGCCGCTCTAT
	IRF7 cDNA R	ACGCACTTCTTACACACCTCC
	PPARG cDNA F	CCAGCGACATCGACCAGTTA
	PPARG cDNA R	TTCCTGCAGTGGTGATGCAT
	HMGA1 cDNA F	AGAACAAGGCCAGCTCCAAA
	HMGA1 cDNA R	GGGTGATGATGAGGTAGCGG
	SMARCB1 cDNA F	GCCCTCTTCTGGAAACCCTC
	SMARCB1 cDNA R	TCCATCTGGGGAGAGGAGTG
